# (μ-Ethane-1,1,2,2-tetra­carboxyl­ato)bis­[tetra­aqua­manganese(II)]

**DOI:** 10.1107/S1600536811008063

**Published:** 2011-03-15

**Authors:** Meng-Wei Xue, Chang-Yun Chen

**Affiliations:** a School of Biochemical and Environmental Engineering, Nanjing Xiaozhuang College, Nanjing 210017, People’s Republic of China

## Abstract

In the centrosymmetric title molecule, [Mn_2_(C_6_H_2_O_8_)(H_2_O)_8_], the Mn^II^ atom is in an octa­hedral environment coordinated by six O-atom donors from water mol­ecules and ethane-1,1,2,2-tetra­carboxyl­ate ligands. The crystal structure features a three-dimensional hydrogen-bonding network based on a strong and distinctive pattern of O—H⋯O hydrogen-bonding inter­actions.

## Related literature

For related literature on metal–organic frameworks, see: Chen *et al.* (2007 ▶); Fan & Zhu (2006 ▶); Li & Yang (2006 ▶). For related literature on hydrogen bonding, see: Forster & Cheetham (2002 ▶); Kim & Jung (2000 ▶).
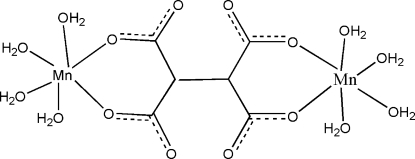


## Experimental

### 

#### Crystal data

[Mn_2_(C_6_H_2_O_8_)(H_2_O)_8_]
*M* *_r_* = 456.08
Triclinic, 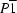

*a* = 6.2901 (12) Å
*b* = 8.0212 (15) Å
*c* = 8.0769 (15) Å
α = 108.522 (3)°
β = 95.068 (3)°
γ = 97.086 (3)°
*V* = 379.92 (12) Å^3^
*Z* = 1
Mo *K*α radiation
μ = 1.75 mm^−1^
*T* = 293 K
0.30 × 0.26 × 0.24 mm

#### Data collection

Bruker SMART CCD area-detector diffractometer
Absorption correction: multi-scan (*SADABS*; Bruker, 2000 ▶) *T*_min_ = 0.785, *T*_max_ = 0.823
1956 measured reflections
1379 independent reflections
1309 reflections with *I* > 2σ(*I*)
*R*_int_ = 0.044

#### Refinement

*R*[*F* ^2^ > 2σ(*F* ^2^)] = 0.026
*wR*(*F* ^2^) = 0.069
*S* = 1.05
1379 reflections
144 parameters
H atoms treated by a mixture of independent and constrained refinement
Δρ_max_ = 0.30 e Å^−3^
Δρ_min_ = −0.30 e Å^−3^

### 

Data collection: *SMART* (Bruker, 2000 ▶); cell refinement: *SMART*; data reduction: *SAINT* (Bruker, 2000 ▶); program(s) used to solve structure: *SHELXTL* (Sheldrick, 2008 ▶); program(s) used to refine structure: *SHELXTL*; molecular graphics: *SHELXTL*; software used to prepare material for publication: *SHELXTL*.

## Supplementary Material

Crystal structure: contains datablocks global, I. DOI: 10.1107/S1600536811008063/pb2059sup1.cif

Structure factors: contains datablocks I. DOI: 10.1107/S1600536811008063/pb2059Isup2.hkl

Additional supplementary materials:  crystallographic information; 3D view; checkCIF report

## Figures and Tables

**Fig. 1.**
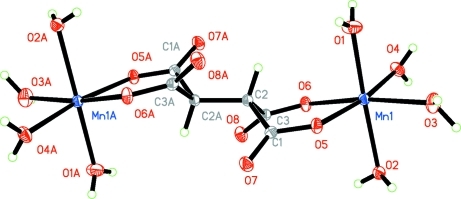
The molecular structure of (I), showing the atom-numbering scheme and displacement ellipsoids at the 30% probability level.

**Fig. 2.**
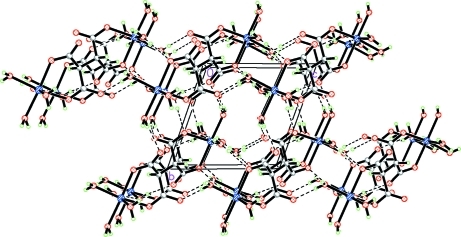
Crystal packing diagram of l.

## supplementary crystallographic information

### Comment

The design and synthesis of metal-organic frameworks(MOFs) has become a very interesting research field. This not only stems from their potential application as functional materials but also from their intriguing structural topologies(Chen *et al.*, 2007; Fan & Zhu, 2006; Li & Yang, 2006). However, frankly speaking, the designed synthesis of coordination networks and supramolecular architectures is still a difficult challenge. The formation of coordination polymers is not only influenced by the geometrical and electronic properties of metal ions but also relying on other factors such as the rigidity or flexibility of the ligands and diversity of metal ions and organic ligands in coordination and noncovalent interactions such as hydrogen bonding (Kim & Jung, 2000; Forster *et al.*, 2002). Therefore, the rational design and construction of coordination polymers based upon assembly of metal ions and multifunctional organic ligands is an interesting research field. Herein we report the crystal structure of the title compound (I).

The molecular structure of (I) is illustrated in Fig. 1., where selected bond distances and bond angles are given in Table 1.

Single-crystal X-ray analysis reveals that 1 crystallizes in the triclinic space group P-1, The structure of 1 is a single molecule in which the asymmetric unit contains one Mn atom, half tce anion, four coordinated water molecules. In complex 1, there is one kind of crystallographically independent Mn^II^ center.1 features a 3-D hydrogen bonding network based on a strong and distinctive pattern of hydrogen bonding interactions. As show in Fig. 2., a one-dimensional chain is formed by bond generated by coordinated water molecule and uncoordinated oxygen atom of ligand tce, Further, one-dimensional chains are linked by bonds to form two-dimensional layers, and two-dimensional layers are also jointed by hydrogen bonds to give rise to three-dimensional structure.

### Experimental

A H_2_O solution (10 ml) of sodium 1,1,2,2-tetracarboxyl-ethylene (29.4 mg, 1 mmol) was added to a CH_3_OH solution (10 ml) of Mn(OAc)_2_2.5H_2_O (17.3 mg,1 mmol). The pH of the mixture was adjusted to about 7. The mixture was stirred for 2 h and then filtered.Single crystals appeared after the filtered solution was allowed to stand at room temperature for 2 days.

### Refinement

The C-bound H atoms were placed to the bonded parent atoms in geometrically idealized positions (C—H = 0.93, and 0.98 Å) and refined as riding atoms, with *U*_iso_(H) = 1.2*U*_eq_(C). The O-bound H atoms were located in difference Fourier maps and refined as riding in their as-found relative positions(O—H =0.96 Å) with *U*_iso_(H) = 1.5*U*_eq_(C).

### Crystal data

**Table:**

[Mn_2_(C_6_H_2_O_8_)(H_2_O)_8_]	*Z* = 1
*M**_r_* = 456.08	*F*(000) = 232.0
Triclinic, *P*1	*D*_x_ = 1.993 Mg m^−^^3^
Hall symbol: -P 1	Mo *K*α radiation, λ = 0.71073 Å
*a* = 6.2901 (12) Å	Cell parameters from 924 reflections
*b* = 8.0212 (15) Å	θ = 2.2–20.2°
*c* = 8.0769 (15) Å	µ = 1.75 mm^−^^1^
α = 108.522 (3)°	*T* = 293 K
β = 95.068 (3)°	Block, brown
γ = 97.086 (3)°	0.30 × 0.26 × 0.24 mm
*V* = 379.92 (12) Å^3^	

### Data collection

**Table:**

Bruker SMART CCD area-detector diffractometer	1379 independent reflections
Radiation source: fine-focus sealed tube	1309 reflections with *I* > 2σ(*I*)
graphite	*R*_int_ = 0.044
φ and ω scans	θ_max_ = 25.5°, θ_min_ = 2.7°
Absorption correction: multi-scan (*SADABS*; Bruker, 2000)	*h* = −6→7
*T*_min_ = 0.785, *T*_max_ = 0.823	*k* = −9→9
1956 measured reflections	*l* = −9→9

### Refinement

**Table:**

Refinement on *F*^2^	Primary atom site location: structure-invariant direct methods
Least-squares matrix: full	Secondary atom site location: difference Fourier map
*R*[*F*^2^ > 2σ(*F*^2^)] = 0.026	Hydrogen site location: inferred from neighbouring sites
*wR*(*F*^2^) = 0.069	H atoms treated by a mixture of independent and constrained refinement
*S* = 1.05	*w* = 1/[σ^2^(*F*_o_^2^) + (0.0451*P*)^2^] where *P* = (*F*_o_^2^ + 2*F*_c_^2^)/3
1379 reflections	(Δ/σ)_max_ = 0.001
144 parameters	Δρ_max_ = 0.30 e Å^−^^3^
0 restraints	Δρ_min_ = −0.30 e Å^−^^3^

### Special details

**Table:**

Geometry. All e.s.d.'s (except the e.s.d. in the dihedral angle between two l.s. planes) are estimated using the full covariance matrix. The cell e.s.d.'s are taken into account individually in the estimation of e.s.d.'s in distances, angles and torsion angles; correlations between e.s.d.'s in cell parameters are only used when they are defined by crystal symmetry. An approximate (isotropic) treatment of cell e.s.d.'s is used for estimating e.s.d.'s involving l.s. planes.
Refinement. Refinement of *F*^2^ against ALL reflections. The weighted *R*-factor *wR* and goodness of fit *S* are based on *F*^2^, conventional *R*-factors *R* are based on *F*, with *F* set to zero for negative *F*^2^. The threshold expression of *F*^2^ > σ(*F*^2^) is used only for calculating *R*-factors(gt) *etc*. and is not relevant to the choice of reflections for refinement. *R*-factors based on *F*^2^ are statistically about twice as large as those based on *F*, and *R*- factors based on ALL data will be even larger.

### Fractional atomic coordinates and isotropic or equivalent isotropic displacement parameters (Å^2^)

**Table:**

	*x*	*y*	*z*	*U*_iso_*/*U*_eq_	
Mn1	0.30762 (4)	0.25813 (4)	0.70315 (3)	0.02040 (14)	
O1	−0.0280 (3)	0.2382 (3)	0.6000 (2)	0.0407 (4)	
O2	0.6555 (2)	0.3118 (2)	0.8276 (2)	0.0249 (3)	
O3	0.3825 (3)	0.5167 (2)	0.6758 (2)	0.0388 (4)	
O4	0.3632 (3)	0.1354 (2)	0.43505 (19)	0.0293 (3)	
O5	0.2172 (2)	0.34158 (18)	0.96515 (17)	0.0238 (3)	
O6	0.2933 (2)	−0.00729 (18)	0.72542 (18)	0.0246 (3)	
O7	0.1900 (2)	0.28929 (19)	1.21579 (17)	0.0282 (3)	
O8	0.3028 (2)	−0.1656 (2)	0.90616 (19)	0.0297 (3)	
C1	0.1621 (3)	0.2406 (2)	1.0530 (2)	0.0186 (4)	
C2	0.0577 (3)	0.0477 (2)	0.9454 (2)	0.0184 (4)	
H9	−0.050 (3)	0.053 (3)	0.853 (3)	0.022*	
C3	0.2319 (3)	−0.0512 (2)	0.8542 (2)	0.0192 (4)	
H3	0.685 (4)	0.421 (5)	0.890 (4)	0.046 (8)*	
H4	0.669 (4)	0.260 (4)	0.883 (4)	0.036 (8)*	
H7	0.292 (4)	0.177 (4)	0.373 (3)	0.034 (7)*	
H8	0.475 (6)	0.111 (5)	0.400 (5)	0.077 (12)*	
H5	0.519 (9)	0.581 (8)	0.691 (7)	0.15 (2)*	
H1	−0.108 (7)	0.278 (6)	0.663 (6)	0.100 (15)*	
H2	−0.092 (6)	0.175 (5)	0.510 (5)	0.067 (11)*	
H6	0.332 (6)	0.591 (6)	0.719 (5)	0.077 (13)*	

### Atomic displacement parameters (Å^2^)

**Table:**

	*U*^11^	*U*^22^	*U*^33^	*U*^12^	*U*^13^	*U*^23^
Mn1	0.0220 (2)	0.02101 (19)	0.01841 (19)	0.00202 (12)	0.00384 (12)	0.00706 (13)
O1	0.0267 (9)	0.0621 (12)	0.0257 (9)	0.0078 (8)	0.0007 (7)	0.0045 (8)
O2	0.0274 (8)	0.0220 (8)	0.0251 (8)	0.0022 (6)	0.0024 (6)	0.0086 (7)
O3	0.0508 (11)	0.0237 (8)	0.0427 (10)	0.0047 (8)	0.0128 (8)	0.0110 (7)
O4	0.0338 (9)	0.0375 (9)	0.0210 (7)	0.0145 (7)	0.0088 (7)	0.0114 (7)
O5	0.0331 (8)	0.0176 (7)	0.0200 (7)	0.0006 (6)	0.0069 (6)	0.0059 (5)
O6	0.0310 (8)	0.0234 (7)	0.0234 (7)	0.0073 (6)	0.0126 (6)	0.0098 (6)
O7	0.0389 (8)	0.0245 (7)	0.0180 (7)	−0.0011 (6)	0.0012 (6)	0.0057 (6)
O8	0.0345 (8)	0.0312 (8)	0.0317 (8)	0.0145 (6)	0.0116 (6)	0.0168 (6)
C1	0.0167 (9)	0.0192 (9)	0.0200 (9)	0.0048 (7)	0.0041 (7)	0.0056 (7)
C2	0.0196 (9)	0.0184 (9)	0.0177 (9)	0.0023 (7)	0.0031 (7)	0.0067 (7)
C3	0.0216 (9)	0.0163 (9)	0.0173 (9)	0.0006 (7)	0.0024 (7)	0.0032 (7)

### Geometric parameters (Å, °)

**Table:**

Mn1—O3	2.1541 (18)	O3—H6	0.71 (4)
Mn1—O4	2.1552 (15)	O4—H7	0.82 (3)
Mn1—O5	2.1578 (13)	O4—H8	0.80 (4)
Mn1—O1	2.1674 (18)	O5—C1	1.273 (2)
Mn1—O6	2.1845 (14)	O6—C3	1.275 (2)
Mn1—O2	2.2557 (16)	O7—C1	1.236 (2)
O1—H1	0.77 (4)	O8—C3	1.237 (2)
O1—H2	0.78 (4)	C1—C2	1.541 (3)
O2—H3	0.85 (3)	C2—C2^i^	1.515 (3)
O2—H4	0.71 (3)	C2—C3	1.536 (2)
O3—H5	0.92 (6)	C2—H9	0.98 (2)
			
O3—Mn1—O4	89.71 (7)	Mn1—O3—H5	126 (3)
O3—Mn1—O5	98.50 (6)	Mn1—O3—H6	123 (3)
O4—Mn1—O5	170.75 (6)	H5—O3—H6	96 (4)
O3—Mn1—O1	90.76 (8)	Mn1—O4—H7	107.7 (18)
O4—Mn1—O1	87.24 (7)	Mn1—O4—H8	128 (3)
O5—Mn1—O1	88.40 (6)	H7—O4—H8	113 (3)
O3—Mn1—O6	169.57 (7)	C1—O5—Mn1	126.22 (12)
O4—Mn1—O6	86.03 (6)	C3—O6—Mn1	125.18 (11)
O5—Mn1—O6	86.55 (5)	O7—C1—O5	123.56 (17)
O1—Mn1—O6	98.54 (7)	O7—C1—C2	120.04 (16)
O3—Mn1—O2	84.27 (8)	O5—C1—C2	116.39 (16)
O4—Mn1—O2	96.98 (6)	C2^i^—C2—C3	112.65 (19)
O5—Mn1—O2	88.12 (6)	C2^i^—C2—C1	112.95 (19)
O1—Mn1—O2	173.45 (7)	C3—C2—C1	108.56 (14)
O6—Mn1—O2	86.79 (6)	C2^i^—C2—H9	107.6 (13)
Mn1—O1—H1	120 (3)	C3—C2—H9	107.4 (13)
Mn1—O1—H2	129 (3)	C1—C2—H9	107.4 (15)
H1—O1—H2	109 (4)	O8—C3—O6	124.24 (16)
Mn1—O2—H3	107.8 (18)	O8—C3—C2	120.15 (16)
Mn1—O2—H4	110 (2)	O6—C3—C2	115.61 (16)
H3—O2—H4	109 (3)		

Symmetry codes: (i) −*x*, −*y*, −*z*+2.

### Hydrogen-bond geometry (Å, °)

**Table:**

*D*—H···*A*	*D*—H	H···*A*	*D*···*A*	*D*—H···*A*
O2—H3···O5^ii^	0.85 (3)	1.88 (3)	2.726 (2)	174 (3)
O2—H4···O8^iii^	0.71 (3)	2.07 (3)	2.765 (2)	166 (3)
O4—H7···O7^iv^	0.82 (3)	1.89 (3)	2.689 (2)	167 (2)
O4—H8···O6^v^	0.80 (4)	1.97 (4)	2.757 (2)	167 (4)
O3—H5···O7^ii^	0.92 (6)	1.95 (6)	2.847 (2)	165 (5)
O1—H1···O2^vi^	0.77 (4)	2.07 (4)	2.818 (2)	164 (5)
O1—H2···O6^vii^	0.78 (4)	2.14 (4)	2.914 (2)	175 (4)
O3—H6···O8^viii^	0.71 (4)	2.10 (4)	2.771 (2)	158 (4)

Symmetry codes: (ii) −*x*+1, −*y*+1, −*z*+2; (iii) −*x*+1, −*y*, −*z*+2; (iv) *x*, *y*, *z*−1; (v) −*x*+1, −*y*, −*z*+1; (vi) *x*−1, *y*, *z*; (vii) −*x*, −*y*, −*z*+1; (viii) *x*, *y*+1, *z*.

## References

[bb1] Bruker (2000). *SMART*, *SAINT* and *SADABS* Bruker AXS Inc., Madison, Wisconsin, USA.

[bb2] Chen, X. N., Zhang, W. X. & Chen, X. M. (2007). *J. Am. Chem. Soc.* **129**, 15738–15739. 10.1021/ja074962i 18044893

[bb3] Fan, S. R. & Zhu, L. G. (2006). *Inorg. Chem.* **45**, 7935–7942. 10.1021/ic060871v 16961387

[bb4] Forster, P. M. & Cheetham, A. K. (2002). *Angew. Chem. Int. Ed.* **41**, 457–459. 10.1002/1521-3773(20020201)41:3<457::aid-anie457>3.0.co;2-w 12491377

[bb5] Kim, Y. J. & Jung, D. Y. (2000). *Inorg. Chem.* **39**, 1470–1475. 10.1021/ic991119f 12526451

[bb6] Li, Y. N. & Yang, R. T. (2006). *J. Am. Chem. Soc.* **128**, 726–727. 10.1021/ja056831s 16417355

[bb7] Sheldrick, G. M. (2008). *Acta Cryst.* A**64**, 112–122. 10.1107/S0108767307043930 18156677

